# St. John's wort extract Ze 117 alters the membrane fluidity of C6 glioma cells by influencing cellular cholesterol metabolism

**DOI:** 10.1038/s41598-024-60562-0

**Published:** 2024-04-30

**Authors:** Swen Bremer, Eva Weitkemper, Hanns Häberlein, Sebastian Franken

**Affiliations:** Institute of Biochemistry and Molecular Biology, Medical Faculty, University of Bonn, Nussallee 11, 53115 Bonn, Germany

**Keywords:** Lipids, Depression

## Abstract

Chronic stress is associated with major depressive disorder (MDD). Increased glucocorticoid levels caused by uncontrolled release through the hypothalamic‒pituitary‒adrenal (HPA) axis can cause changes in the lipid content of the cellular plasma membrane. These changes are suspected to be involved in the development of depressive disorders. St. John's wort extract (SJW) Ze 117 has long been used as an alternative to synthetic antidepressants. Part of its effect may be due to an effect on the cellular lipid composition and thus on the properties of plasma membranes and receptor systems embedded therein. In this study, we investigated the effect of Ze 117 on that of dexamethasone and simvastatin. Dexamethasone increases the fluidity of C6 cell plasma membranes. This effect is counteracted by administration of Ze 117. Here we demonstrate that this is not due to a change in C16:1/16:0 and C18:1/18:0 ratios in C6 cell fatty acids. On the other hand, Ze 117 increased the cellular cholesterol content by 42.5%, whereas dexamethasone reduced cholesterol levels similarly to simvastatin. Lowering cholesterol levels by dexamethasone or simvastatin resulted in decreased β-arrestin 2 recruitment to the 5-HT_1a_ receptor. This effect was counterbalanced by Ze 117, whereas the SJW extract had little effect on β-arrestin 2 recruitment in non-stressed cells. Taken together, in C6 cells, Ze 117 induces changes in membrane fluidity through its effect on cellular cholesterol metabolism rather than by affecting fatty acid saturation. This effect is reflected in an altered signal transduction of the 5-HT_1a_ receptor under Ze 117 administration. The current in vitro results support the hypothesis that Ze 117 addresses relevant parts of the cellular lipid metabolism, possibly explaining some of the antidepressant actions of Ze 117.

## Introduction

Depression is a very common mental disease that occurs in all age groups, from childhood to elderlyhood, and comes in many different levels of severity. It has been shown that the prevalence for adults experiencing depression throughout their lives is approximately 12.9% worldwide^1^. Although depression is widespread, little is known about its causes, making it difficult to choose the right treatment for the patient and increasing the need for further research. Factors that play a role in the development of depression include genetic inheritance as well as various environmental influences, including social isolation, witnessing traumatic events and experiencing chronic stress^2^. On a biochemical basis, several theories have been introduced over the years aiming to explain and understand the underlying mechanisms of depression. These include the monoamine hypothesis, monoaminergic receptor hypothesis, signaling hypothesis and neuroplasticity hypothesis^3–7^. However, due to the complexity of this disease, many questions remain unanswered.

Another hypothesis states that chronic stress may lead to an overactivity of the hypothalamic‒pituitary‒adrenal (HPA) axis, resulting in an uncontrolled release of the stress hormone cortisol^8,9^. Increased glucocorticoid levels, in turn, can cause changes in the plasma membrane lipid composition^10^. These changes, for example, in brain cells, also seem to be involved in the development of depressive disorders^11^. It has been shown that the lipid composition of cellular membranes can affect the interaction and function of receptors/proteins found within them and thus their signaling ability^12,13^. Measurable physical properties of the plasma membrane, such as its fluidity, can also be determined by the lipid composition, particularly the desaturation status of their fatty acid moieties and the cholesterol content^14,15^. Several independent research groups have shown that exposure of different cell types to glucocorticoids results in an increase in their membrane fluidity, suggesting a close link between a hyperactive HPA axis, changes in plasma membrane lipid composition and depression^16–18^. It has been shown that increased membrane fluidity causes decreased binding of serotonin to its receptor in mouse brain membranes^19^. Furthermore, increased brain membrane fluidity was found in other mental disorders, such as in the prefrontal cortices of patients suffering from schizophrenia^20^. There is also evidence that Ze 117, a St. John’s wort (SJW) extract, which has been used for several decades to treat mild to moderate depression, can counteract the glucocorticoid-induced increase in membrane fluidity of C6 cells^18^. In addition, the SJW ingredient hyperforin was shown to modify neuronal membrane properties in vivo^21^. Therefore, stabilizing the plasma membrane fluidity of brain cells after excessive exposure to glucocorticoids could offer a possible explanation for the molecular mechanism of action of Ze 117 in the treatment of depressive disorders. However, the cellular mechanisms behind the opposing effect of SJW and glucocorticoids on membrane fluidity have not been satisfactorily elucidated.

Based on our previous work, we hypothesize that Ze 117 induces changes in membrane fluidity by influencing relevant pathways of the cellular lipid metabolism^18,22^, because the degree of saturation of lipids contained in plasma membranes as well as the cholesterol content of membranes are two essential parameters for the regulation of membrane fluidity^23,24^, we used a proteomics approach focusing on cellular lipid metabolism to investigate changes in the expression of desaturases, especially stearoyl-CoA desaturase (SCD), which is the rate-determining enzyme in the conversion of saturated to unsaturated fatty acids, and enzymes of cholesterol metabolism. The latter brings into focus the synthesis of cholesterol for the membrane fluidity changing effect of Ze 117.

## Results

### Dexamethasone and Ze 117 influence the mRNA level of stearoyl-CoA desaturase 1 in C6 cells

We have shown in previous work that Ze 117 alters the membrane fluidity of C6 cells, most likely by affecting the cellular lipid composition^18^. To identify potential targets of Ze 117 within the cellular lipid metabolism that could explain the observed effect on the membrane fluidity of C6 cells, we performed a whole-cell proteomic analysis after pretreatment of the cells with Ze 117.

An SJW extract concentration of 25.0 µg/ml was identified in previous work as effective but not toxic to C6 cells^18^. In total, we were able to detect and identify 4375 individual proteins confidently. Furthermore, we checked whether the enzymes involved in the lipid metabolism processes of interest were among the proteins found in the identification and quantification processes. To our satisfaction, all processes of interest were well represented by our study (see SI Table 1).

Next, we tested the effect of Ze 117 on the abundances of the enzymes involved in these lipid metabolism processes compared to the untreated control with a special focus on desaturases and cholesterol metabolism. None of the identified desaturases (stearoyl-CoA desaturase 2, acyl-CoA (3–8)-desaturase, acyl-CoA 6-desaturase) were significantly regulated by Ze 117 (SI Table 1).

Unfortunately, owing to the lack of individual values, the data for stearoyl-CoA desaturase 1 (SCD1), which is the other isoform of this rate-determining enzyme in the conversion of saturated to unsaturated fatty acids, were not meaningful. Nevertheless, because of the central role of SCD1 in fatty acid synthesis, we decided to determine the amount of its mRNA in C6 cells by quantitative PCR. In addition, we compared a possible influence of Ze 117 to dexamethasone, which shows a similar effect on the membrane fluidity of C6 cells as demonstrated for cortisol (Additional file 1).

**Figure 1:**
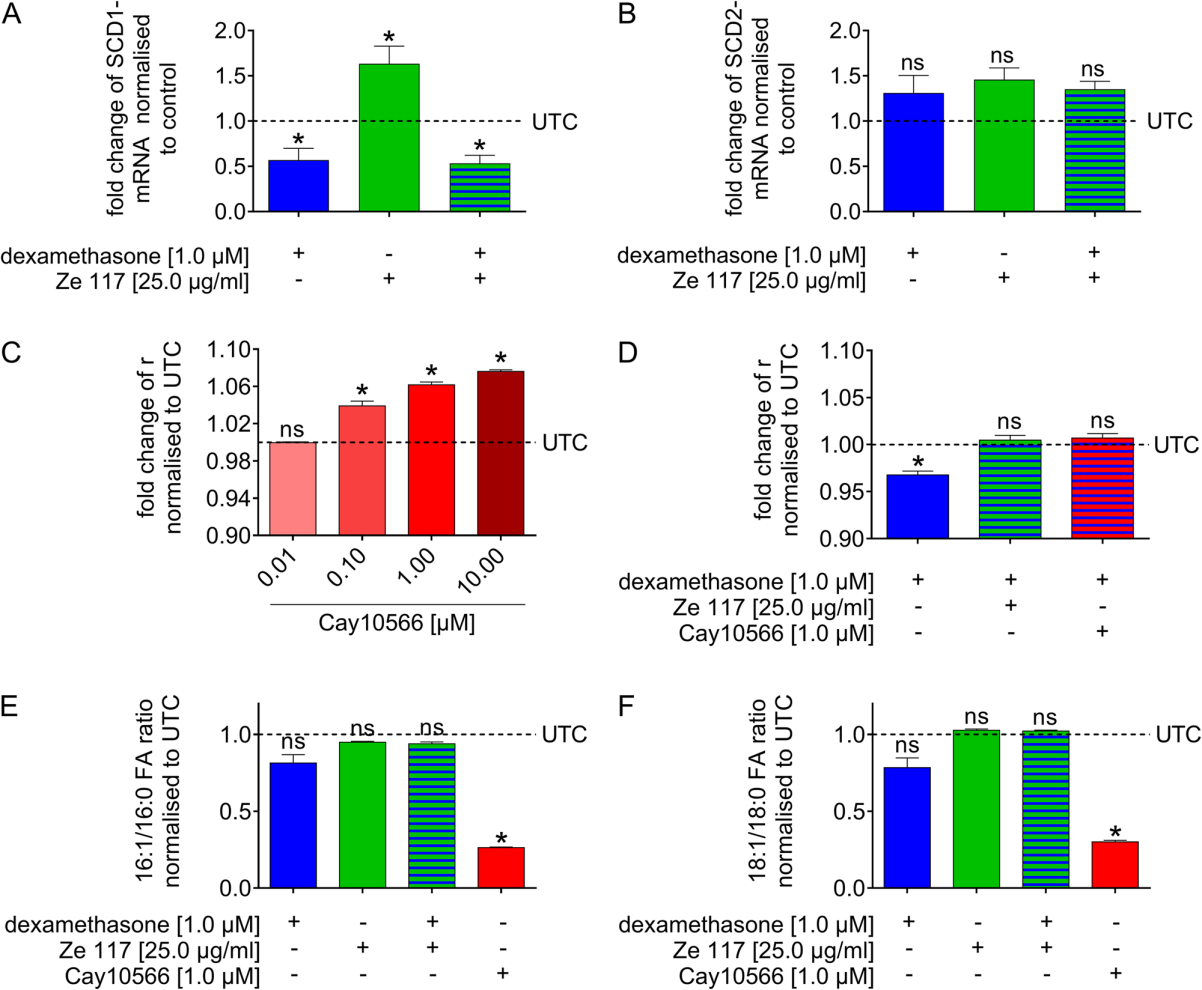
Effect of the SCD1 inhibitor Cay10566, Ze 117, dexamethasone and combinations of Cay10566/dexamethasone and Ze 117/dexamethasone on a variety of parameters. Relative fold change of SCD1 (**A**) and SCD2 (**B**) mRNA levels in C6 cells pretreated with dexamethasone, Ze 117, and their combination for 48 h compared to untreated control (UTC) cells. Relative mRNA fold changes were calculated by using a modified version of the Pfaffl equation with actin beta (Actb), ribosomal protein L21 (RPL21) and ribosomal protein L13 (Rpl13) as reference genes. Relative fold change in DPH fluorescence anisotropy measured in the plasma membrane of C6 cells in suspension after a 48 h preincubation with (**C**) different concentrations of the SCD inhibitor CAY10566 compared to untreated control cells (UTC) and (**D**) either dexamethasone (1.0 µM) alone or in combination with Ze 117 (25.0 µg/ml) or CAY10556 (1.0 µM). The effect of dexamethasone and Ze 117 as a single treatment and in combination on the ratio of (**E**) palmitate (C16:0) and palmitoleate (C16:1) as well as of (**F**) stearate (C18:0) and oleate (C18:1) after 48 h of treatment compared to the effect of the SCD inhibitor Cay10566. The relative fatty acid abundance values of each test condition were set into a ratio and normalized to the mean ratio of the respective untreated control (UTC). All results shown in this figure are displayed as the mean and SEM of at least three individual experiments. Values marked * were considered significantly different (unpaired t test with Welch’s correction) compared to the untreated control with p ≤ 0.05.

When incubated with dexamethasone (1.0 µM) alone, SCD1 mRNA levels were decreased significantly by 0.569 ± 0.130-fold (mean ± SEM) (Fig. 1A). Interestingly, single treatment with Ze 117 increased SCD1 mRNA levels significantly by 1.633 ± 0.195-fold (mean ± SEM). In contrast to the normalizing effect of Ze 117 on the dexamethasone-mediated changes in membrane fluidity (Additional file 1), the dexamethasone-mediated decrease in SCD1 mRNA level was not abolished by Ze 117 when coincubated for 48 h (Fig. 1A). Interestingly, none of the conditions tested showed a significant effect on SCD2 mRNA levels under the same treatment conditions, which fits the results from our protein analysis (Fig. 1B; SI Table 1).

### Inhibition of stearoyl-CoA desaturase 1 counteracts dexamethasone induced changes in membrane fluidity

Since both test substances had a significant effect on the SCD1 mRNA level in C6 cells, we next investigated whether affecting SCD1 can cause measurable effects on plasma membrane fluidity and thus explain the observed effect of dexamethasone and/or Ze 117 on the plasma membrane of C6 cells.

For this purpose, C6 cells were incubated with 0.01, 0.1, 1, and 10 µM of the specific SCD1 inhibitor Cay10566 for 48 h, and fluorescence anisotropy measurements were performed. Three out of the four tested inhibitor concentrations significantly increased the C6 cell plasma membrane fluorescence anisotropy of the probe DPH in a dose-dependent manner by 1.036 ± 0.001-fold (mean ± SEM) for 0.1 µM, 1.059 ± 0.002-fold (mean ± SEM) for 1.0 µM and 1.076 ± 0.001-fold (mean ± SEM) for 10 µM (Fig. 1C) compared to the untreated control, indicating a decrease in plasma membrane fluidity. Next, we tested whether the glucocorticoid-mediated increase in membrane fluidity of C6 cells could be counteracted by concomitant treatment with the SCD1 inhibitor Cay10566 (1.0 µM) or Ze 117 (25.0 µg/ml) (Fig. 1D). When coincubated, Cay10566 and Ze 117 similarly counteracted the decrease in relative fluorescence anisotropy caused by dexamethasone, from 0.970 ± 0.001-fold (mean ± SEM) to 1.007 ± 0.002-fold (mean ± SEM) and 0.999 ± 0.002-fold (mean ± SEM), respectively, compared to the untreated control. This represents normalization of the C6 cell plasma membrane fluidity.

### Ze 117 does not change the activity of stearoyl-CoA desaturase 1 in C6 cells

Since SCD1 offers a plausible target in the regulation of plasma membrane fluidity and dexamethasone as well as Ze 117 both alter SCD1 mRNA levels, it was next tested whether this alteration directly affects SCD1 activity in C6 cells by determining the ratios of the SCD1 products and educts.

Despite the mRNA changes, incubation of C6 cells with dexamethasone for 48 h did not significantly alter the fatty acid desaturation indices C16:1/16:0 and C18:1/18:0, with values of 0.813 ± 0.054-fold (mean ± SEM) and 0.784 ± 0.063-fold (mean ± SEM), respectively, compared to their untreated controls (Fig. 1E and F). Interestingly, the same phenomenon was observed for Ze 117. The increase in SCD1 mRNA content did not change the catalytic activity of the enzyme SCD either. The values for the fatty acid indices C16:1/16:0 and C18:1/18:0 were 0.948 ± 0.006-fold (mean ± SEM) and 1.027 ± 0.008-fold (mean ± SEM), respectively. As expected, incubating the cells with the SCD1 inhibitor (Cay10556) led to significantly decreased fatty acid indices of 0.263 ± 0.004-fold (mean ± SEM) for C16:1/16:0 and 0.301 ± 0.010-fold (mean ± SEM) for C18:1/18:0 compared to the untreated control, indicating a measurable inhibition of the enzymatic activity of SCD1 (Fig. 1E and F).

### Ze 117 increases cellular cholesterol content in C6 cells

In addition to the degree of fatty acid saturation of plasma membrane lipids, cholesterol content is a very important cellular tool to regulate and maintain the physiological fluidity of cellular membranes. When incubated with Ze 117, 10 out of the 47 identified proteins involved in lipid metabolism processes were considered significantly upregulated. Interestingly, all of these belong to the cholesterol biosynthesis cascade (SI Table 1). To examine whether the detected upregulation of several enzymes involved in cholesterol biosynthesis results in a significant increase in cellular cholesterol content in C6 cells, the effect of Ze 117 on the total cholesterol content relative to the untreated control was investigated after a 48 h preincubation with Ze 117.

**Figure 2:**
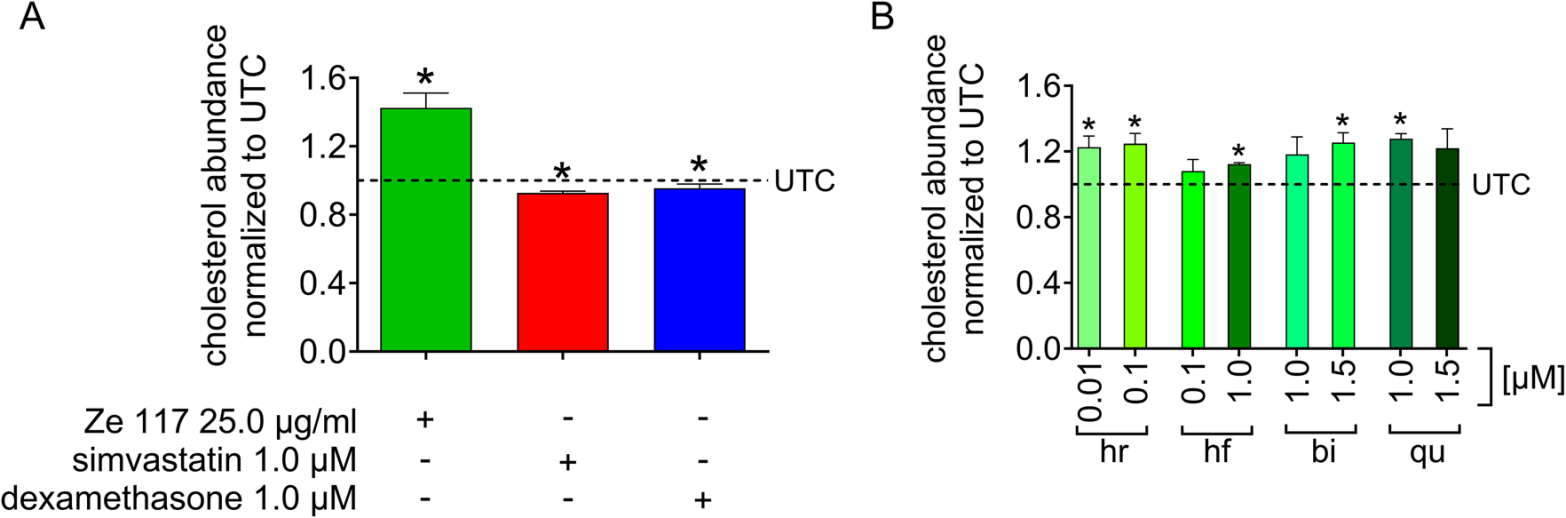
Effect of single Ze 117 ingredients on the total cholesterol content of C6 cells. (**A**) Relative fold change in total cholesterol content in C6 cells after 48 h of preincubation with Ze 117 (25.0 µg/ml), simvastatin (1.0 µM) or dexamethasone (1.0 µM) compared to untreated control (UTC) cells. (**B**) Relative fold change in total cholesterol content in C6 cells after 48 h of preincubation with hypericin (hr; 0.01 and 0.1 µM), hyperforin (hf; 0.1 and 1.0 µM), biapigenin (bi; 1.0 and 1.5 µM) or quercetin (qu; 1.0 and 1.5 µM) compared to the untreated control. The results are displayed as the mean and SEM of at least three individual experiments. Values marked * were considered significantly different (unpaired t test with Welch’s correction) compared to the untreated control (UTC) with p ≤ 0.05.

As shown in Fig. 2A, 25.0 µg/ml Ze 117 significantly increased the total cholesterol content of C6 cells by 1.425 ± 0.086-fold (mean ± SEM). Treating the cells with the cholesterol biosynthesis inhibitor simvastatin (1.0 µM) caused a significant decrease in the total cellular cholesterol content by 0.927 ± 0.011-fold (mean ± SEM) compared to the untreated control. Dexamethasone also decreased the total cellular cholesterol content 0.955 ± 0.011-fold (mean ± SEM) compared to the untreated control.

Ze 117 contains different classes of ingredients, such as flavonoids (quercetin, biapigenin), naphthodianthrones (hypericin), and phloroglucinol derivatives (hyperforin), for which it was previously shown that they affect the membrane fluidity of C6 cells^18^. We therefore tested these single compounds at the same concentrations as in the work of Keksel et al.^18^ for their ability to affect the cellular cholesterol content. Biapigenin at 1.5 µM as well as quercetin and hyperforin at 1 µM all showed a significant increase in cholesterol content, with hyperforin showing the lowest increase (1.125 ± 0.004-fold (mean ± SEM)) and quercetin the highest (1.278 ± 0.018-fold (mean ± SEM)) compared to the untreated control. Hypericin affected cholesterol levels at much lower concentrations, leading to a significant increase of 1.226 ± 0.040-fold (mean ± SEM) at a concentration as low as 0.01 µM (Fig. 2B).

**Figure 3:**
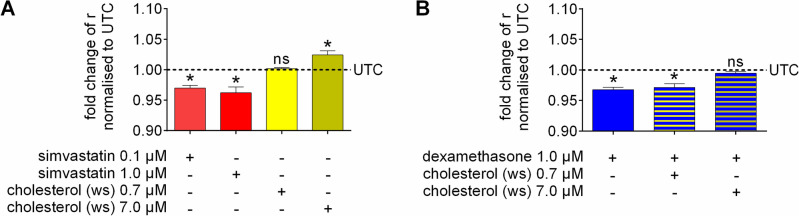
Effect of cholesterol on the fluidity of the C6 cell plasma membrane. Relative fold change of fluorescence anisotropy measured in the plasma membrane of C6 cells in suspension after a 48 h preincubation with (**A**) different concentrations of cholesterol biosynthesis inhibitor simvastatin and water soluble cholesterol compared to untreated control cells (UTC) and (**B**) either dexamethasone (1.0 µM) alone or in combination with water soluble cholesterol (0.7 µM and 7.0 µM). The results are displayed as the mean and SEM of at least three individual experiments. Values marked * were considered significantly different (unpaired t test with Welch’s correction) compared to the untreated control (UTC) with p ≤ 0.05.

To evaluate whether alterations in the cellular cholesterol content could be responsible for changes in membrane fluidity of C6 cells as seen for Ze 117 in our experimental setup, we pretreated the cells with either simvastatin (0.1 or 1.0 µM, respectively) or water-soluble cholesterol (MβCD-cholesterol) (0.7 or 7.0 µM, respectively) for 48 h and performed fluorescence anisotropy experiments (Fig. 3). Simvastatin led to a significant decrease in fluorescence anisotropy, with values of 0.970 ± 0.003-fold (mean ± SEM) when using 0.1 µM and 0.962 ± 0.006-fold (mean ± SEM) when using 1.0 µM (Fig. 3A). Increasing the amount of cholesterol available to the cells by the addition of 7.0 µM water soluble cholesterol to the cell culture medium caused a significant increase in fluorescence anisotropy by 1.024 ± 0.004-fold (mean ± SEM) compared to the untreated control. The tenfold lower concentration (0.7 µM) did not have an effect, with a value of 1.002 ± 0.001-fold (mean ± SEM) (Fig. 3A). Since changing the cellular content of cholesterol has proven to be a membrane fluidity regulating factor, we tested whether the dexamethasone-mediated effect on membrane fluidity could be counteracted by increasing the amount of cholesterol available to the cells, similar to the effect seen for Ze 117. The simultaneous incubation of the cells with 7.0 µM water soluble cholesterol for 48 h successfully reversed the effect of dexamethasone (1.0 µM) (0.995 ± 0.003-fold (mean ± SEM)). On the other hand, 0.7 µM had no effect (Fig. 3B).

### Cholesterol content affects 5-HT_1A_ receptor signaling in transfected HEK 293 cells

To investigate whether the impairment of cholesterol biosynthesis and thus the resulting changes in cellular cholesterol content have an effect on the functionality of receptors known to be involved in depression, the effect of simvastatin, water soluble cholesterol, and Ze 117 on β-arrestin 2 recruitment after 5-HT_1A_-receptor stimulation with serotonin was tested in transiently transfected HEK 293 cells.

**Figure 4:**
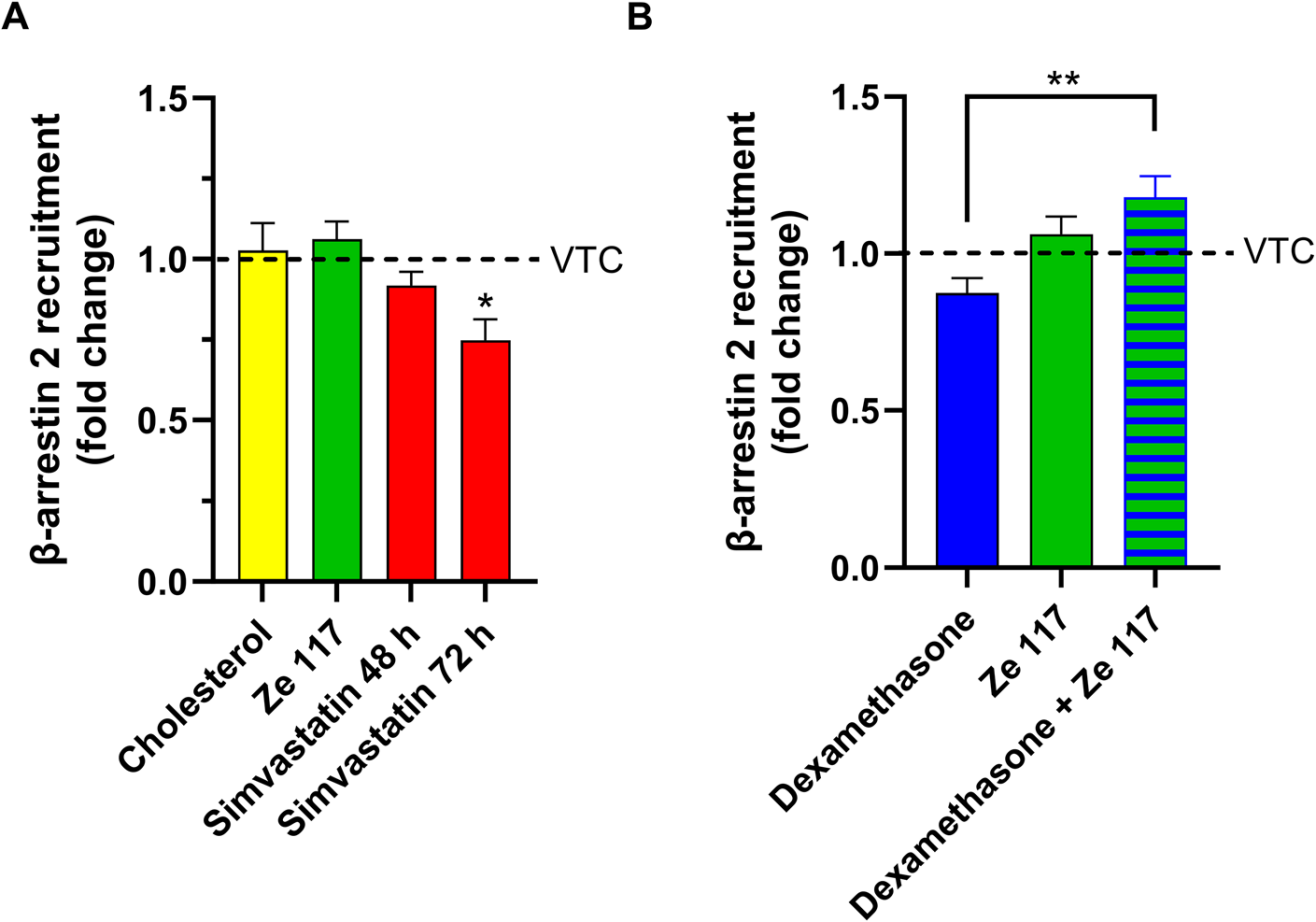
Effect of cholesterol content on β-arrestin 2 recruitment to the 5-HT_1a_ receptor. (**A**) 5HTR_1a_ NanoBiT-βarr2 HEK 293 cells were preincubated with 7.0 µM water soluble cholesterol, 25.0 µg/ml Ze 117, or 1.0 µM simvastatin for 48 h or 1.0 µM simvastatin for 72 h before stimulation with 5.0 µM serotonin, and luminescence was measured for 60 min. The area under the curve (AUC) was calculated from four independent experiments. Every substance is displayed in relation to its vehicle-treated control (VTC). Values are given as the means ± SEMs. *p < 0.05 values were significantly different (one-way analysis of variance, Dunnett’s post hoc) compared to VTC. (**B**) 5HTR_1a_ NanoBiT-βarr2 HEK 293 cells were preincubated with 1.0 µM dexamethasone, 25.0 µg/ml Ze 117, or a combination of both for 48 h before stimulation with 5.0 µM serotonin, and luminescence was measured for 60 min. The areas under the curves (AUCs) were calculated from three independent experiments. Every substance is displayed in relation to its vehicle-treated control (VTC). Values are given as the means ± SEMs. **p < 0.01 values were significantly different (one-way analysis of variance, Dunnett’s post hoc).

Whereas single treatment of the cells for 48 h with 7.0 µM water soluble cholesterol (1.027 ± 0.085 (mean ± SEM)) or 25.0 µg/ml Ze 117 (1.062 ± 0.055 (mean ± SEM)) had no or a slightly increasing effect on the recruitment of β-arrestin 2, 1.0 µM simvastatin caused a reduction by 0.918 ± 0.042-fold and 0.662 ± 0.033-fold (mean ± SEM) after 48 and 72 h, respectively, compared to the untreated control (Fig. 4A).

Based on our initial observation that Ze 117 primarily acts on the plasma membrane fluidity of cells stressed with corticosteroids such as dexamethasone, we next tested whether this effect was also observed at the level of 5-HT_1a_ receptor activity. Incubation with dexamethasone resulted in a slight decrease in the recruitment of β-arrestin 2 by the receptor, whereas Ze 117 tended to increase recruitment, as seen previously. Interestingly, this enhancing effect of SJW extract was much more pronounced and statistically significant in cells stressed with dexamethasone than in stressed cells without Ze 117 coincubation (Fig. 4B).

## Discussion

Among the standard drug-related therapeutic approaches for the treatment of depressive disorders, St. John’s wort (SJW) is one of the most recognized herbal alternatives that can be as effective as synthetic drugs such as selective serotonin reuptake inhibitors (SSRIs) but with fewer side effects^25,26^. It has not only been successfully used for decades in this field but is also recommended by the “World Federation of Societies of Biological Psychiatry” (WFSBP), “Canadian Network for Mood and Anxiety Treatments Taskforce” (CANMAT) and German National Health Care Guideline for Unipolar depression as the treatment of choice for mild to moderate depression^27,28^. The majority of the clinical studies mentioned in the meta-analysis by Linde et al. 2005/2008 were conducted with hyperforin-rich extracts. For our preclinical analyses, we opted for the low-hyperforin extract Ze 117, as it was shown by Zahner et al. 2019 that there are no clinically relevant interactions with cytochrome P450 enzymes and P-glycoprotein^29^. Several theories on the molecular mode of action of SJW have been developed over the years, including alterations in neurotransmitter levels in the brain^30^, affecting 5-HT and β-adrenergic receptor movements and densities^31,32^ and affecting the function of the HPA axis^33^. In addition, different activities have been demonstrated for some ingredients of SJW. Hyperforin, for example, activates TRPC6 channels and modulates neuronal plasticity^34,35^. Hypericin, another component of SJW, shows a positive effect on neurotrophin signaling and the formation of gap junctions in various animal models of depression^36,37^. Unfortunately, despite these efforts, none of these hypotheses are able to fully reveal in detail the complete molecular mode of action of SJW.

Recently, our research group was able to demonstrate that the in vitro incubation of C6 and PBMC cells with the stress hormone cortisol increased their plasma membrane fluidity^18,22,32^. Interestingly, this glucocorticoid-mediated increase was neglected by concomitant incubation of the cells with Ze 117 in both cases. As a basis for the effect of Ze 117 on membrane fluidity, the data collected in the C6 cell and PBMC studies indicate changes in lipid metabolism in general and in the ratio of saturated fatty acids (SFA) to monounsaturated fatty acids (MUFA) in plasma membrane lipids, also known as the ‘membrane fluidity index’, in particular^38^. The enzyme responsible for the conversion of SFAs, mainly palmitate and stearate, into their corresponding MUFAs, palmitoleate and oleate, is stearoyl-CoA desaturase (SCD). It has been shown in previous reports that an enhanced expression/activity of SCD can lead to an increased MUFA level in plasma membranes and thus result in increased membrane fluidity and additionally changed other cellular properties^39,40^. This finding fits our observation that the inhibition of SCD1 by the specific SCD1 inhibitor Cay10566 significantly decreased the membrane fluidity of C6 cells in a dose-dependent manner, similar to what was previously observed for other cell types^41^. Furthermore, Cay10566 had the same counteracting effect on the dexamethasone-mediated membrane fluidity increase, as shown for Ze 117. However, although we detected effects of both dexamethasone and Ze 117 on the mRNA levels of SCD1, these changes did not result in corresponding changes in the C16:1/16:0 and C18:1/18:0 ratios for either treatment. Alterations in the expression of SCD mRNA without having an effect on fatty acid desaturation indices have already been demonstrated in another context^42^. Therefore, it can be assumed that the effect of dexamethasone or Ze 117 that we observed is not due to an SCD-mediated change in the membrane desaturation index.

In addition to the degree of fatty acid desaturation, the cholesterol content is reported to have the greatest influence on the fluidity of cell membranes^23,24^. Our proteomic approach revealed that Ze 117, when incubated with C6 cells for 48 h, led to an increased abundance of several proteins involved in the biosynthesis of cholesterol, while the other lipid metabolism-related processes remained unaffected. Of the 18 identified proteins attributable to cholesterol biosynthesis, 10 were significantly upregulated by Ze 117. This is particularly interesting because an increase in cholesterol content in membranes is related to decreased fluidity^15,43^. On the other hand, a decrease in cholesterol abundance in the plasma membrane has been found to increase plasma membrane fluidity^44^. Therefore, increasing the amount of cholesterol in the plasma membrane by activating cellular cholesterol biosynthesis might play a role in the molecular mode of action of Ze 117 in regard to regulating plasma membrane fluidity.

To test the effect of decreased and increased cholesterol contents on the plasma membrane fluidity of C6 cells, we performed a series of fluorescence anisotropy experiments. Our data clearly show that the inhibition of HMG-CoA-reductase, the rate-limiting enzyme of cholesterol biosynthesis, by simvastatin led to decreased fluorescence anisotropy values and thus increased plasma membrane fluidity. On the other hand, an increased availability of cholesterol resulted in increased fluorescence anisotropy, accounting for a decrease in membrane fluidity. Our data also show that the dexamethasone-mediated increase in membrane fluidity can be fully reversed by increasing the amount of cholesterol available to the cells. These results demonstrate that Ze 117 induced elevation of cholesterol biosynthesis, and thus, increased cellular cholesterol levels could be accountable for its membrane fluidity stabilizing effect.

Since our results indicated a possible connection between the cellular cholesterol content, plasma membrane fluidity and Ze 117 treatment, we next investigated the effect of the extract on the cholesterol content of C6 cells. Our results clearly show that Ze 117 significantly increased the cellular cholesterol content when applied for 48 h. To our knowledge, we are the first research group to have investigated the effect of St. John’s wort on the cellular cholesterol content of C6 cells. We therefore hypothesize that the membrane fluidity stabilizing effect of Ze 117 might be mediated, at least in part, by affecting the cholesterol content of C6 cells. To further investigate which of the major constituents of Ze 117 contributes most to the cholesterol-increasing effect, C6 cells were treated with hypericin, hyperforin, biapigenin or quercetin, which represent the main compound classes in SJW extracts. The ingredients and concentrations used were chosen in accordance with the publication by Keksel et al.^18^. All individual compounds caused a significant increase in the total cholesterol content of the cells after 48 h of incubation. Considering the concentrations, hypericin is the most effective of the tested ingredients, while hyperforin has the least influence on cellular cholesterol levels. The observed effect of the single Ze 117 compounds fits well with the results of Keksel et al.^19^ regarding membrane fluidity. Interestingly, none of the individual substances increased the cholesterol content to the same extent as Ze 117. Of the substances studied, quercetin has already been shown to affect cellular cholesterol metabolism. In contrast to our observations, it was demonstrated that quercetin causes a reduction in cellular cholesterol in C6 cells rather than an increase^45^. However, in this study, up to 20 times the amount of quercetin with shorter incubation times compared to our approach was applied, which could explain the observed differences. Comparable data for hypericin, hyperforin and biapigenin were not found in the literature. We also investigated the effect of dexamethasone on the cellular cholesterol content. Our results demonstrate that dexamethasone reduces the total cholesterol content in C6 cells, which is consistent with previous findings^46^. As expected, inhibition of HMG-CoA reductase by simvastatin also reduced the total cholesterol found in C6 cells, indicating the reliability of the experimental setup. Interestingly, Glombik et al.^47^ found a reduction in sterol regulatory element-binding protein (SREBP-2), a regulator of cholesterol biosynthesis, but no change in SCD in the hippocampi of WKY rats, an animal model for depression, fitting perfectly to the results in our study.

Membrane fluidity dysregulation has been shown to interfere with the functionality of membrane-bound receptors. For example, it was recently shown that alterations in membrane fluidity are involved in the inhibition of granulocyte–macrophage colony-stimulating factor receptor^48^. Additionally, changes in plasma membrane fluidity alter the ligand binding activity of G protein-coupled receptors (GPCRs)^49^. Moreover, cholesterol not only affects membrane fluidity but also has the ability to directly bind scaffold proteins such as NHERF1, which are heavily involved in cellular signaling^50^. The 5-HT_1A_ receptor is a GPCR for which involvement in depression pathology has been shown in multiple studies^51^. In addition, its functionality was demonstrated to be dependent on the cellular cholesterol content^47,52^. We therefore investigated whether Ze 117, simvastatin, or dexamethasone not only change the total cellular cholesterol content but also affect the signaling ability of this receptor. Our data demonstrate that decreasing the amount of cellular cholesterol by simvastatin clearly led to decreased recruitment of the scaffold protein β-arrestin 2 to the 5-HT_1A_ receptor. This observation is consistent with the results of Kumar and Chattopadhyay, who demonstrated decreased internalization of the 5-HT_1A_ receptor, which at least in part depends on the recruitment of β-arrestin, under statin treatment^53^. Furthermore, for other GPCRs, such as lysophosphatidic acid receptor LPA1, decreased β-arrestin recruitment under cholesterol depletion could also be demonstrated^54^. Remarkably, increasing the cholesterol content under standard cell culture conditions, for example, by Ze 117, had no comparable effect on β-arrestin 2 recruitment to the 5-HT_1A_ receptor. In contrast, cells stressed by dexamethasone reacted to Ze 117 by a significant increase in β-arrestin 2 recruitment. This finding fits the observation that reduction of membrane fluidity by Ze 117 is much more pronounced in cells that were stressed with corticosteroids^18^.

## Conclusion

The data presented here lead to the conclusion that the molecular mode of antidepressant action of St. John’s wort extract Ze 117 may be based in part on its effect on cellular cholesterol metabolism rather than by affecting fatty acid saturation. Several of the tested single compounds in Ze 117 are partly responsible for this effect. Since none of the individual substances tested reached the effect level of Ze 117, it can be speculated that the treatment with the total extract over that with one ingredient alone is more effective. The data point to a new mechanism of action of Ze 117 that needs to be verified in further in vivo studies.

## Methods

### Chemicals

Unless otherwise stated, all chemicals were purchased from Sigma (Taufkirchen, Germany). Coelenterazine h (HPLC purity > 95%) was purchased from Prolume Ltd. (Pinetop, AZ, United States).

Hypericin (HPLC purity 99.09%), biapigenin (HPLC purity 99.72%), hyperforin (HPLC purity 99.56%, including 17.20% adhyperforin), and quercetin (HPLC purity 96%) were purchased from PhytoLab GmbH & Co. KG (Vestenbergsgreuth, Germany).

Max Zeller Söhne AG in Romanshorn (Switzerland) provided the *Hypericum perforatum* dried extract Ze 117 (DER 4–7:1; extraction solvent ethanol 57.9% (V/V); batch number 16113301) with 0.26% hypericin and < 0.2% hyperforin. The extract was manufactured according to Ph. Eur. EP 9.3/1874. The dried extract was redissolved in 50% ethanol to a final concentration of 50 mg/ml and used as a stock solution.

### General cell culture

C6 wild-type cells obtained from the German Collection of Microorganisms and Cell Culture GmbH (DSMZ; Braunschweig, Germany) were cultivated on 10 cm cell culture plates in fully supplemented DMEM/F12 medium (DMEM/F12 medium, 5% FCS, 1% GLUTAMAX (100×), penicillin (100 U/ml), streptomycin (0.1 mg/ml) in an incubator at 37 °C, 5% CO_2_ and high humidity. Cells were passaged every 3 days at a ratio of 1:20.

### Proteomic analysis

#### Cell culture and cell lysis

Approximately 6 × 10^4^ C6 wild-type cells were seeded into one well of a 6-well cell culture plate and either incubated with St. John’s wort extract Ze 117 (25.0 µg/ml) or the corresponding amount of 50% ethanol (Ze 117 solvent) acted as a solvent control for 48 h in an incubator at 37 °C, 5% CO_2_ and high humidity. The culture medium was then removed, the cells were washed at least three times with PBS, and cell lysis was performed directly in each well by adding 150 µl of sodium deoxycholate (SDC) lysis buffer containing 5% SDC, 0.4 µM triethylammonium bicarbonate, HALT Protease Inhibitor Cocktail (Thermo Fisher Scientific, Rockford, USA) and 5 mM ethylenediaminetetraacidic acid. Cell lysates were each transferred from their culture plate to a 1.5 ml Eppendorf tube, and DNA was sheared using a BIORUPTOR Plus sonication device equipped with a Minichiller 300 (Diogenode, Seraing, Belgium). From this step onwards, cell lysates were either kept on ice or frozen at −20 °C. The total protein content in each sample was finally determined by performing a BCA Protein Assay (PIERCE, Thermo Fisher Scientific, Rockford, USA) according to the user manual. For each test condition, four independent replicates were prepared.

#### Protein digestion and peptide purification

For protein digestion, approximately 50 µg of protein from each cell lysate sample was transferred to a VWR centrifuge filter (VWR International, Pennsylvania, USA). For reduction, proteins were incubated with the reducing agent dithiothreitol (20 mM) for 30 min at 55 °C. After the reduction reaction was completed, the reduced proteins were washed twice with 400 µl of working buffer (0.5% SDC and 2% tetraethylammonium bromide dissolved in water). Alkylation was performed by incubating the proteins with 100 µl of a 40 mM acrylamide solution at room temperature (RT) for 30 min. Again, samples were washed twice with 100 µl working buffer. Protein digestion was performed by incubating the reduced and alkylated protein samples with the enzyme trypsin at 37 °C for 12 h and constant shaking in an Eppendorf ThermoMixer C (Eppendorf GmbH, Wesseling, Germany). The resulting peptide fragments were eluted from the filter, and the detergent SDC was removed by precipitation with formic acid (FA) and extraction with ethyl acetate. Finally, the solvent was removed by vacuum evaporation using an Eppendorf Concentrator plus (Eppendorf, Hamburg, Germany) connected to a diaphragm pump (Vacuubrand GmbH + Co, Wertheim, Germany).

#### TMT labeling and peptide purification

For simultaneous mass spectrometry analysis, each peptide sample was individually labeled with a specific TMTpro 16 plex label reagent (Thermo Fisher Scientific, Rockford, USA) according to the user manual provided with the kit. Afterwards, 10% of each labeled peptide sample was combined into two pools, and salt contaminants from the labeling process were removed by passing this complex peptide mixture across an OASIS HLB 1 cc Flangeless cartridge (Waters GmbH, Eschborn, Germany). The solvent was removed by vacuum evaporation, and the dried peptides were stored at −20 °C until further use. Samples were redissolved in 20 mM ammonium formate (pH 10) and fractionated by reversed-phase chromatography at elevated pH with a Reprosil 100 C18 column (3 µm 125 × 4 mm, Dr. Maisch GmbH, Ammerbuch-Entringen, Germany). Sixty fractions were combined into 6 pools and dried in a vacuum concentrator^55^. Peptides were purified by solid phase extraction (Oasis HLB cartridges, Waters GmbH, Eschborn, Germany).

#### LC/MS measurements

LC/MS measurements and data processing were performed as described in the publications of Karen Pollecker et al., Lies van Baarle et al., and Naila Umer et al.^55–57^. Before measurement, peptides were redissolved in 0.1% FA, and 1 µg was separated on a Dionex Ultimate 3000 RSLC nano HPLC system (Dionex GmbH, Idstein, Germany). The autosampler was operated in μl-pickup mode. A C18 analytical column was made in-house (400 mm length, 75 µm inner diameter, REPROSIL-PUR 120 C18-AQ, 1.9 µm, Dr. Maisch). Peptides were separated during a linear gradient from 5 to 35% solvent B (90% acetonitrile (ACN), 0.1% FA) at 300 nL/min for 110 min. The nanoHPLC was coupled online to an Orbitrap Fusion Lumos mass spectrometer (Thermo Fisher Scientific, Bremen, Germany). Peptide ions between 330 and 1600 m/z were scanned in the Orbitrap detector every 3.5 s with a resolution of 120,000 (maximum fill time 50 ms, AGC target 50%). Polysiloxane (445.1200 Da) was used for internal calibration (typical mass error ≤ 1.5 ppm). In a top-speed method, peptides were subjected to collision-induced dissociation for identification (CID: 0.7 Da isolation, normalized energy 30%), and fragments were analyzed in a linear ion trap with an AGC target of 100% and a maximum fill time of 35 ms in rapid mode. Fragmented peptide ions were excluded from repeat analysis for 30 s. The top 10 fragment ions were chosen for synchronous precursor selection and fragmented with higher energy CID (HCD: 3 Da MS2 isolation, 65% collision energy) for detection of reporter ions in the Orbitrap analyzer (range 100–200 m/z, resolution 50,000, maximum fill time 86 ms, AGC target 200%).

#### Data processing

Raw data processing and database search were performed with Proteome Discoverer software 2.5.0.400 (Thermo Fisher Scientific). Peptide identification was performed with an in-house Mascot server version 2.6.1 (Matrix Science Ltd, London, UK). MS data were searched against sequences from the UniProt reference proteome rat (2021/04) and contaminants database (cRAP^1^). The precursor ion m/z tolerance was 10 ppm, and fragment ion tolerance was 0.5 Da (CID). Tryptic peptides with up to two missed cleavages were searched. Propionamide-modification of cysteines and TMTpro on N-termini and lysines were set as static modifications. Oxidation was allowed as a dynamic modification of methionine. Mascot results were evaluated by the Percolator algorithm version 3.05^2^ as implemented in Proteome Discoverer. Spectra with identifications above 1% q-value were sent to a second round of database search with semi tryptic enzyme specificity (one missed cleavage allowed). Protein N-terminal acetylation, methionine oxidation, TMTpro, and cysteine alkylation were then set as dynamic modifications. Actual FDR values were 0.6% (peptide spectrum matches) and 1.0% (peptides). Reporter ion intensities (most confident centroid) were extracted from the MS3 level, with SPS mass match > 65% and coisolation threshold of 90%.

#### Statistical analysis

For statistical analysis, the measured protein abundances for all test conditions and replicates of a single protein were normalized to the corresponding averaged untreated control protein abundance. Proteins that increased or decreased by 10% or more compared to the untreated control were considered significantly different if the p value calculated by an unpaired t test with Welch’s correction was ≤ 0.05.

### Fluorescence anisotropy measurements

C6 cells were seeded into a 10 cm cell culture plate and pretreated with a variety of test compounds (Ze 117 [25.0 µg/ml]; cortisol, corticosterone and dexamethasone [1.0 µM]; Cay 10,566 [0.01, 0.1, 1.0 and 10.0 µM] simvastatin [0.1 and 1.0 µM]; *Methyl*-β-*Cyclodextrin (MβCD)*-Cholesterol [0.7 and 7.0 µM]; various combinations) for 48 h. On the day of the experiment, cells were detached from the plate, counted, resuspended in Hank’s balanced salt solution (HBSS), and adjusted to 2 × 10^6^ cells/ml. Next, the fluorescent probe diphenylhexatriene (DPH) was added to a final concentration of 2.5 µM, and the cell suspensions were incubated for 30 min at 37 °C. For measurement, 2 ml of each cell suspension was transferred into a cuvette, which was then inserted into the temperature-controlled cuvette holder of the fluorescence spectrometer LS 55 (Perkin-Elmer, Rodgau, Germany) and kept there for 5 min prior to the measurements. The emission polarizer was fixed to a vertical position, while the absorption polarizer could switch between the vertical and horizontal positions. If both polarizers were positioned vertically (vv), polarized light signals were measured parallel. If, on the other hand, the absorption polarizer changed its position from vertical to horizontal (vh), polarized light signals were measured perpendicularly. The excitation wavelength was set to 360 (± 5) nm, and the emission wavelength was set to 430 (± 5) nm. Throughout the measurement, the cell suspension was constantly stirred by a magnetic stirring device and maintained at 37 °C. For each test condition, ten individual measurements were recorded per experiment. The experiment was repeated at least three times. The fluorescence anisotropy r was calculated based on the measured light intensities (I_vv_) and (I_vh_) using the following equation:

1 $${\mathrm{Anisotropy\, r}}= \frac{{\text{Ivv}}-\mathrm{ Ivh}}{{\text{Ivv}}+2 \times \mathrm{ Ivh}}$$

### RT‒qPCR experiment

For each test condition, approximately 4 × 10^4^ C6 cells were seeded into one well of a 12-well cell culture plate and incubated for 48 h with dexamethasone (1.0 µM) and SJW extract Ze 117 (25.0 µg/ml), individually and in combination. The concentrations of 1.0 µM of the corticosteroids and 25.0 µg/ml of Ze 117 were chosen based on previous publications^18,22,32^. Next, C6 cell RNA was extracted and purified according to the manufacturer’s user manual of the NucleoSpin RNA plus XS kit (Macherey–Nagel GmbH & Co. KG, Düren, Germany). The total RNA amount of each test condition was evaluated using a NANODROP instrument (Thermo Scientific, Waltham, MA, USA). The mRNA expression levels of the two genes of interest, stearoyl-Coa desaturase 1 and 2 (SCD1 and 2), as well as of three reference genes (beta-actin [Actb], 60S ribosomal protein L21 [RPL21], and 60S ribosomal protein L13 [RPL13]), were measured by performing a real-time quantitative polymerase chain reaction (RT‒qPCR) using the Power SYBR GREEN RNA-to C_T_ 1–Step Kit (Thermo Fisher Scientific, Vilnius, Lithuania) on a CFX OPUS 96 Dx PCR cycler (Bio-RAD, Hercules, CA, USA). For that purpose, approximately 5 ng of the total RNA extract was used for each test condition. A melting curve analysis was performed to ensure the specificity of the resulting qPCR products. Whether the treatments had a significant effect on the mRNA expression levels of SCD1 and 2 compared to the untreated control was tested by calculating their relative gene expression using two reference genes and a modified version of the Pfaffl equation (Eq. 2). All values were normalized to the mean of the untreated control. Statistically significant differences between the treatments and the untreated control were determined by performing an unpaired t test with Welch’s correction. All measurements were performed at least in duplicate, and the experiments were repeated three times as biological replicates. The sequences of the primer pairs used are shown in Table 1.

**Table 1:** Primers.

Target	Primer sequences	
Stearoyl-CoA-Desaturase 1 (SCD1)	Forward	5′-gaa cac cca tcc ggt gag aca g-3′
	Reverse	5′-tct gca ggt ttc cgg aag ga-3′
Stearoyl-CoA-Desaturase 2 (SCD2)	Forward	5′-ctc tct gca cgt tct cat ccc-3′
	Reverse	5′-att tca ggg cga aca tct gct-3′
Ribosomal Protein L21 (RPL21)	Forward	5′-tac cat ggc aaa acc gga aga g-3′
	Reverse	5′-gga agc tgt ctc tgc tct ttg a-3′
Beta-actin (Actb)	Forward	5′-tgc agt ttc tgc tct ttc cca g-3′
	Reverse	5′-ggg agc gcg taa ccc tca ta-3′
Ribosomal Protein L13 (RPL13)	Forward	5′-taa att ggc cac gca gct aac ag-3′
	Reverse	5′-tgc tct gcg gct ttc ttt cg-3′

2 $${\mathrm{Relative\, gene\, expression}}=\frac{{({{\text{E}}}_{{\text{GOI}}})}^{\Delta {\mathrm{Ct\, GOI}}}}{{{{\text{GeoMean}}[({\text{E}}}_{{\text{REF}}})}^{\Delta {\mathrm{Ct\, REF}}}]}$$

### SCD activity by carbon-16 and carbon-18 desaturation indices

C6 cells were either treated solitarily with dexamethasone (1.0 µM), St. John’s wort extract Ze 117 (25.0 µg/ml) and SCD inhibitor Cay10566 (1.0 µM) or in combination with dexamethasone and Ze 117 for 48 h. Next, the culture medium was removed, and the cells were washed three times with phosphate buffered saline (PBS) and lysed in 100 µl distilled water using a BIORUPTOR Plus sonication device equipped with a Minichiller 300 (Diogenode, Seraing, Belgium). For acidic hydrolysis, 100 µl of the total cell lysate was mixed in a glass vial with 900 µl ACN containing H_2_SO_4_ (0.75 M) and butylhydroxytoluene (50 µg/ml) to achieve a final H_2_O/ACN ratio of 1:9 (v/v). To start the chemical reaction, the samples were heated to 90 °C for 5 h while constantly being stirred. The samples were then allowed to cool to room temperature, and free fatty acids were extracted by adding 2.25 ml n-hexane and vigorous vortex-mixing for 5 min. The fatty acid-containing n-hexane layer was transferred into a clean glass vial (La-Pha-Pack GmbH, Langerwehe, Germany), and the extraction process was repeated. Both obtained fatty acid fractions were combined, and the solvent was removed by vacuum evaporation. The previously described procedure for fatty acid hydrolysis and MS analysis has already been postulated before for the fatty acid analysis of human plasma samples^58^.

For MS analysis, dried fatty acid samples were resuspended in 100 µl methanol/H_2_O (1:1, v/v) and directly injected into an Orbitrap Fusion Lumos Tribrid Mass Spectrometer (Thermo Fisher Scientific, San Jose, USA), which was controlled by Thermo FreeStyle 1.8 SP2 software (Thermo Fisher Scientific, Waltham, USA). Fourier transform MS analysis was performed in negative ion mode for a m/z range of 200 to 500. For each test condition and replicate, an individual MS scan was recorded, and relative fatty acid abundances were calculated for the fatty acids of interest. The calculated abundances of the monounsaturated fatty acids (MUFAs) palmitoleate (16:1) and oleate (18:1) were set into ratios with their corresponding saturated fatty acids (SFAs) palmitate (16:0) and stearate (18:0) of the same sample and MS scan, resulting in fatty acid desaturation indices of 16:1/16:0 and 18:1/18:0. For all test conditions, both indices were calculated, normalized and compared to the mean of the untreated control. At least three individual experiments were performed (biological replicates), and significant differences between the untreated control and the treatments were identified by performing an unpaired t test with Welch’s correction.

### Quantification of C6 cell total cholesterol

For each test condition, an appropriate amount of C6 wild-type cells was seeded into 3 wells of a 96-well plate and incubated for 48 h with Ze 117 (25.0 µg/ml), simvastatin (1.0 µM), dexamethasone (1.0 µM), hypericin (0.01 and 0.1 µM), hyperforin (0.1 and 1.0 M), biapigenin (1.0 and 1.5 µM) or quercetin (1.0 and 1.5 µM). Ethanol and methanol, as solvents, were added to the wells chosen for the untreated control condition. At the end of the 48-h preincubation, total cellular cholesterol content was determined using the Cholesterol/Colesterol ester-Glo Assay (Promega GmbH, Mannheim, Germany) according to the manufacturer’s protocol. To account for differences in cholesterol levels between test conditions resulting from differences in cell count, the mean cell count of each test condition was determined, and a correction factor was calculated. For this purpose, the cell suspensions used to seed the cells for the cholesterol quantification assay were also seeded into another 96-well plate and treated identically. At the end of the 48-h preincubation period, the medium was removed carefully, and the cells were washed two times with warm (37 °C) phosphate-buffered saline (PBS). For fixation, cells were incubated with a 4% paraformaldehyde (PFA) solution in PBS for 15 min at 37 °C. Next, the PFA solution was removed carefully, and the cells were washed three times with PBS. Triton (0.1% in PBS) was applied for 3 min, and the cells were washed with PBS three times. For DNA staining, the fluorescent DNA probe Draq 5 was diluted 1:10,000 in PBS, and 50 µl was applied to each well. Cells were incubated for 1 h at 37 °C. Cells were washed three times with PBS, and fluorescence was measured at 700 nm using an Odyssey lx fluorescence scanner (LI-COR Biosciences, Lincoln, USA). The cell count-corrected cholesterol levels were normalized to the untreated control. Normalized cholesterol changes from three individual experiments (biological replicates) were used for statistical analysis. Significance was tested by an unpaired t test with Welch’s correction. Test conditions with P values ≤ 0.05 were considered significantly different from the untreated control (UTC).

### NanoBiT-β-arrestin 2 recruitment assay

HEK 293 cells (DSMZ; Braunschweig, Germany) were cultivated in DMEM with a low glucose concentration supplemented with 10% (v/v) FBS and 100 IU/mL penicillin. For each independent experiment, HEK 293 cells were transiently transfected with pcDNA3_5HT1a-LgBiT and pcDNA3.1Zeo_SmBiT-β-Arrestin2 using polyethylenimine. Twenty-four hours after transfection, the cells were seeded (20,000 cells/well) into white 96-well plates with clear bottoms (Greiner, Kremsmünster, Austria). The culture medium of the cells was replaced 24 h later with medium containing test compounds and incubated for 48 h or 72 h at 37 °C and 5% CO_2_. An appropriate solvent control was performed for each test compound. After incubation, the culture medium was removed from the cells and replaced with 45 µL of HBSS/HEPES containing 2.5 µM coelenterazine h. The plate was incubated for 7 min at 37 °C and 5% CO_2_ and then transferred to a SPARK multimode microplate reader (Tecan, Männedorf, Switzerland) set to 30 °C. One reading was taken per minute. After 5 cycles, the measurement was interrupted, and 5 µL buffer or a tenfold concentrated ligand solution was added. The measurement was continued for 55 more cycles. At least three independent experiments were performed (biological replicates), and analysis of the raw data was performed as described by Saecker et al.^59^. To detect significant differences between the different cell treatments, normalized AUC data of β-arrestin 2 recruitment were examined by analysis of variances (ANOVA), followed by a post hoc test (Dunnett's).

### Supplementary Information

Supplementary Information.

## Data Availability

The datasets used and/or analyzed during the current study are available from the corresponding author upon reasonable request.

## Abbreviations

5-HT_1a_ receptor: Serotonin 1A receptor
CANMAT: Canadian Network for Mood and Anxiety Treatments Taskforce
DPH: Diphenylhexatriene
HMG-CoA: 3-Hydroxy-3-Methylglutaryl-Coenzym-A
HPA: Hypothalamic‒pituitary‒adrenal axis
MDD: Major depressive disorders
MUFA: Monounsaturated fatty acids
MβCD: *Methyl*-β-*Cyclodextrin*
PBMC: Peripheral blood mononuclear cells
SCD: Stearoyl-CoA desaturase
SFA: Saturated fatty acids
SJW: St. John's wort extract
SREBP: Sterol regulatory element-binding protein
SSRI: Selective serotonin reuptake inhibitors
WFSBP: World Federation of Societies of Biological Psychiatry
