# Supplementary material for: St. John's wort extract Ze 117 alters the membrane fluidity of C6 glioma cells by influencing cellular cholesterol metabolism
Source: Sci Rep. 2024 Apr 30;14:9878. doi: 10.1038/s41598-024-60562-0 (PMC11059309; doi:10.1038/s41598-024-60562-0)
Supplement: Supplementary file 1 — Supplementary Information. [file 41598_2024_60562_MOESM1_ESM.pdf]

# St. John's wort extract Ze 117 alters the membrane fluidity of C6 glioma cells by influencing cellular cholesterol metabolism

Swen Bremer[1], Eva Weitkemper[1], Hanns Häberlein[1] and Sebastian Franken[1,*]

[1]Institute of Biochemistry and Molecular Biology, Medical Faculty, University of Bonn, Nussallee 11, 53115 Bonn, Germany; sbremer1@uni-bonn.de (S.B.); s6evweit@uni-bonn.de (E.W.); haeberlein@uni-bonn.de (H.H.)

*Correspondence: sfranken@uni-bonn.de (S.F.)

**SI Table 1: Effect of Ze 117 on the abundance of proteins involved in lipid metabolism in C6 cells.**

| Protein name | Gene symbol | Biological Process | Ze 117 25 µg/ml |
|---|---|---|---|
| Stearoyl-CoA desaturase 2 | SCD2 | Desaturation of FA | ↔ |
| Acyl-CoA (3-8)-desaturase | FADS1 | | ↔ |
| Acyl-CoA 6-desaturase | FADS2 | | ↔ |
| Elongation of very long-chain fatty acids protein 1 | Elovl1 | Elongation of FA | ↔ |
| Very-long-chain 3-oxoacyl-CoA reductase | Hsd17b12 | | ↔ |
| Very-long-chain (3R)-3-hydroxyacyl-CoA-dehydrogenase | Hacd3 | | ↔ |
| Very-long-chain enoyl-CoA reductase | Tecr | | ↔ |
| Fatty acid synthase | Fasn | Fatty acid synthesis | ↔ |
| Very-long-chain specific axyl-CoA dehydrogenase | Acadvl | Beta-oxidation of FA | ↔ |
| Long-chain specific axyl-CoA dehydrogenase | Acadl | | ↔ |
| Medium-chain specific acyl-CoA dehydrogenase | Acadm | | ↔ |
| Short-chain specific acyl-CoA dehydrogenase | Acads | | ↔ |
| Enoyl-CoA hydratase | Echs1 | | ↔ |
| Hydroxyacyl-CoA dehydrogenase | Hadh | | ↔ |
| Acetyl-CoA acetyltransferase 1 | Acat1 | | ↔ |
| Enoyl-CoA Delta Isomerase 1 | Eci1 | | ↔ |
| Enoyl-CoA Delta Isomerase 1 | Eci2 | | ↔ |

| | | | |
|---|---|---|---|
| Acetyl-CoA acetyltransferase 1 | Acat1 | Cholesterol biosynthesis | ↔ |
| Hydroxymethyl-glutaryl-CoA Synthase 1 | HMGCS1 | | ↔ |
| 3-hydroxy-3-methylglutaryl-CoA reductase | HMGCR | | ↔ |
| Mevalonate kinase | Mvk | | ↔ |
| Phospho-mevalonat kinase | Pmvk | | ↑ |
| Diphospho-mevalonate decarboxylase | Mvd | | ↑ |
| Isopentenyl-diphosphate Delta-isomerase | IDI | | ↔ |
| Farnesyl pyrophosphate synthase | Fdps | | ↑ |
| Squalene synthase | Fdft1 | | ↑ |
| Squalene monooxygenase | Sqle | | ↑ |
| Lanosterol synthase | Lss | | ↑ |
| Lanosterol 14-alpha demethylase | Cyp51a1 | | ↔ |
| Delta(24)-sterol reductase | Dhcr24 | | ↑ |
| Delta(14)-sterol reductase | Lbr | | ↔ |
| Methylsterol momooxygenase | Msmo1 | | ↔ |
| Sterol-4-alpha-carboxylate 3-dehydrogenase | Nsdhl | | ↑ |
| 3-keto-steroid reductase | Hsd17b7 | | ↑ |
| 7-dehydro-cholesterol reductase | Dhcr7 | | ↑ |
| Glycerol kinase | Gk | Phospholipid synthesis | ↔ |
| Glycerol-3-phosphate dehydrogenase | Gpd2 | | ↔ |

| | | |
|---|---|---|
| Long-chain-fatty-acid CoA ligase 1 | Acsl1 | ↔ |
| Long-chain-fatty-acid CoA ligase 3 | Acsl3 | ↔ |
| Long-chain-fatty-acid CoA ligase 4 | Acsl4 | ↔ |
| Long-chain-fatty-acid CoA ligase 5 | Acsl5 | ↔ |
| Glycerol-3-phosphate acyltransferase 3 | Gpat3 | ↔ |
| Glycerol-3-phosphate acyltransferase 4 | Gpat4 | ↔ |
| Choline-phosphate cytidylyl-transferase | Pcyt1a | ↔ |
| Ethanolamine-phosphate cytidylyl-transferase | Pcyt2 | ↔ |
| Phosphatidyl-serine synthase 1 | Ptdss1 | ↔ |
| Lyso-phosphatidyl-choline acyltransferase 3 | Lpcat3 | ↔ |

**Ze 117: St John´s Wort extract; ↑: Protein significantly upregulated compared to untreated control; ↓: Protein significantly downregulated compared to untreated control; ↔: Protein abundance is not changed compared to untreated control**

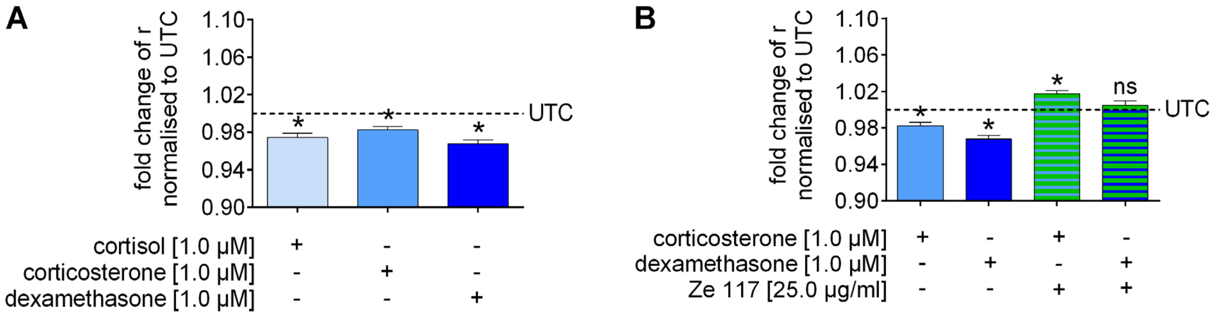


**Additional file 1: Effect of glucocorticoids alone and in combination with Ze 117 on the membrane fluidity of the C6 cell plasma membrane.**

Relative fold change in fluorescence anisotropy measured in the plasma membrane of suspended C6 cells after a 48 h preincubation with (A) 1.0 µM cortisol, corticosterone, or dexamethasone compared to untreated control cells (UTC) and (B) either corticosterone or dexamethasone with or without Ze 117 compared to untreated control cells (UTC). The results are displayed as the mean and SEM of at least three individual experiments. Values marked * were considered significantly different (unpaired t test with Welch´s correction) compared to the untreated control with $p \leq 0.05$.
